# Development and application of a dynamic transmission model of health systems’ preparedness and response to COVID-19 in twenty-six Latin American and Caribbean countries

**DOI:** 10.1371/journal.pgph.0000186

**Published:** 2022-03-08

**Authors:** Adrián Santoro, Alejandro López Osornio, Ivan Williams, Martín Wachs, Cintia Cejas, Maisa Havela, Ariel Bardach, Analía López, Federico Augustovski, Andrés Pichón Riviere, Adolfo Rubinstein

**Affiliations:** 1 Center for Implementation and Innovation in Health Policies, Institute for Clinical Effectiveness and Health Policy, Autonomous City of Buenos Aires, Argentina; 2 Faculty of Economics, University of Buenos Aires, Autonomous City of Buenos Aires, Argentina; 3 Department of Health Technology Assesments (HTA) and Health Economics, Institute for Clinical Effectivenessand Health Policy, Autonomous City of Buenos Aires, Argentina; Babcock University, NIGERIA

## Abstract

The global impact of COVID-19 has challenged health systems across the world. This situation highlighted the need to develop policies based on scientific evidence to prepare the health systems and mitigate the pandemic. In this scenario, governments were urged to predict the impact of the measures they were implementing, how they related to the population’s behavior, and the capacity of health systems to respond to the pandemic. The overarching aim of this research was to develop a customizable and open-source tool to predict the impact of the expansion of COVID-19 on the level of preparedness of the health systems of different Latin American and the Caribbean countries, with two main objectives. Firstly, to estimate the transmission dynamics of COVID-19 and the preparedness and response capacity of health systems in those countries, based on different scenarios and public policies implemented to control, mitigate, or suppress the spread of the epidemic. Secondly, to facilitate policy makers’ decisions by allowing the model to adjust its parameters according to the specific pandemic trajectory and policy context. How many infections and deaths are estimated per day?; When are the peaks of cases and deaths expected, according to the different scenarios?; Which occupancy rate will ICU services have along the epidemiological curve?; When is the optimal time increase restrictions in order to prevent saturation of ICU beds?, are some of the key questions that the model can respond, and is publicly accessible through the following link: http://shinyapps.iecs.org.ar/modelo-covid19/. This open-access and open code tool is based on a SEIR model (Susceptible, Exposed, Infected and Recovered). Using a deterministic epidemiological model, it allows to frame potential scenarios for long periods, providing valuable information on the dynamics of transmission and how it could impact on health systems through multiple customized configurations adapted to specific characteristics of each country.

## Introduction

The global impact of COVID-19 has challenged health systems across the world. The public health threat it represents is the most serious seen in a respiratory virus since the 1918 H1N1 influenza pandemic. Since the beginning of the COVID-19 epidemic in China, warnings have been made about the ability of the SARS-CoV-2 virus to spread and its possible impact in different countries [1–3].This situation showed the necessity to develop policies based on scientific evidence to prepare the health systems to mitigate the pandemic.

By July 2021, SARS-Cov-2 worldwide reported cases reached one hundred and ninety million cases and more than four million deaths. Latin America and the Caribbean (LA&C) is one of the regions with the highest death toll, and almost all countries faced a second and a third wave by the time this paper was being written.

As different countries are taking different public health measures to face the pandemic, it is necessary to predict the extent to which their healthcare systems are prepared to respond to this challenge, since they have become hotspots of the COVID-19 pandemic, exacerbated by weak social protection, fragmented health systems, and profound inequalities.

According to the World Bank, the health crisis sharply contracted regional growth, with a GDP decline of 6.7 percent [4]. This has had an enormous social and economic impact, given that it occurred after several years of slow economic growth and limited progress in social indicators.

Several health policies, like hygiene, masks and social distancing, non-essential business and schools’ closure, self-isolation for risk groups, whole-of-society quarantine, or testing-tracing-isolation strategies, have been implemented with different degrees of success. On the other hand, vaccines are becoming increasingly available during 2021, although access and vaccination rates are very heterogeneous among different countries.

The first published models showed the potential consequences of the expansion of the epidemic through dynamic mathematical models of transmission, commonly used to estimate the spread of infectious diseases. They highlighted the risks of not implementing adequate mitigation and control policies, leaving the transmission dynamics to its natural evolution [5,6]. In this scenario, governments were urged to predict the impact of the policies they were about to implement, their link with the population’s behavior, and the capacity and resilience of health systems to respond to the pandemic.

In 2020, the Institute for Clinical Effectiveness and Health Policy, through the Center for Implementation and Innovation in Health Policy and the Health Technology Assessment and Economic Evaluation Department, developed a model to estimate the impact of the expansion of COVID-19 on the level of preparedness of the health systems of different Latin American and the Caribbean countries. This project, supported by the Interamerican Development Bank (IBD), had two main objectives: 1) to estimate the transmission dynamics of COVID-19 and predict the preparedness and response capacity of health systems in Latin American and Caribbean countries, based on different scenarios and public policies implemented in each country to control, mitigate or suppress the spread of the epidemic, and 2) to facilitate policy makers’ decisions, by allowing a fluid interaction with the model adjusting its parameters according to the specific pandemic trajectory and policy context in each country, and therefore predicting the potential impact of public health interventions to allow healthcare systems to respond timely and appropriately. The 26 countries included in the projections were the so-called Regional Member Countries which, as of June 30, 2020, had about two million cases of COVID-19 reported in their epidemiological surveillance systems and about 120000 deaths, representing a cumulative incidence of approximately 3100 cases per million population and a mortality rate of 180 deaths per million population.

In this paper we describe the development process of this model and its characteristics.

## Methods

The COVID-19 preparedness and response model estimate the impact of the pandemic on the health systems of 26 Latin American and Caribbean countries. It includes epidemiological, health resources, and public policy data from the selected countries. It is oriented towards decision-makers and technical analysts of national or subnational governments in the region. Using a deterministic epidemiological model, allows to frame potential scenarios for long periods [7], providing valuable information on the dynamics of transmission and the impact on health systems through multiple customized configurations adapted to specific characteristics of each country. The different stages for model building (from conceptualization to programming, calibration, validation, transparency, and reporting) followed international modeling good practices for models in general [8–11] and dynamic modeling [12]. Our model is fed with updated daily country-specific COVID-19 mortality statistics to calibrate the pandemic dynamics and better estimate its future trajectory.

The open-access and open code tool is based on a SEIR model (Susceptible, Exposed, Infected and Recovered), commonly used to study the transmission dynamics of infectious diseases [13–16].

This model divides the population in four compartments: "susceptible" (S), "exposed" (E), "infected" (I) and "recovered" (R). To be able to stratify by clinical severity, infected individuals are also grouped into "mild" (I), "severe" (Is) and "critical" (Ic) cases. Recovered individuals can be "immunized" or "deceased."

The COVID-19 preparedness and response model describe the trajectory of the epidemic per day *t*, where the number of individuals in each compartment-state is given by the following system:

$$S_{t}=S_{t-1}-S_{t-1}\alpha$$

$$E_{t}=E_{t-1}+S_{t-1}\alpha -E_{t-1}\gamma$$

$$I_{t}=I_{t-1}+E_{t-1}\gamma -I_{t-1}\delta$$

$$R_{t}=R_{t-1}+I_{t-1}\delta$$

$$N=S_{t}+E_{t}+I_{t}+R_{t}$$

The transition rates from S to E, from E to I and from I to R, are expressed with the Greek letters *α*, *γ* and δ. The first one is:

$$\alpha =R_{0}\delta \frac{I_{t-1}}{N_{t-1}}$$

The last two are modeled as the inverse of the average duration *λ*_*E*_ and *λ*_*I*_ in each compartment E and I, and all of them are assumed as constant. Duration on state I comes from the weighted average duration that each infected subject stays in the mild, severe, and critical stages.

Our model supports two mechanisms to fit existing data: 1) in countries where individual death records with age-group data are available, the total number of infected cases for that particular country is calculated based on age-specific infectious fatality rates (IFR) arising from countries’ seroprevalence surveys when available, or systematic reviews published in peer-review journals, and 2) where only summary death counts are available, with no age information, a global IFR value is applied to all deaths.

In addition, the model performs simulations that start from an initial state (17 days prior to the model update). The total number of individuals in compartments S, E, I and R are estimated as follows: the quantity of new infected (*i*_*t*_) in the first 17 days of the simulation is inferred from the deaths that occurred 17 days later (length of the interval for critical cases) and the infectious fatality rate (f), according to the following formula:

$$i_{t}=\frac{d_{t+17}}{f}.$$

This methodology assumes that there is less underreport for deaths than they are for cases, that it is less affected by the country testing strategy, and that fatality remains constant throughout the pandemic (although there is some evidence of the possible underestimation of the deaths, this evidence is heterogeneous and non-conclusive, without known direction and size). From the estimate of daily new cases described in the previous paragraph, the rest of the compartments are estimated forthe first 17 days of the simulation, except for the stock of exposed:

$$E_{t}=i_{t+1}*\lambda_{E}$$

In this stage (where it is not possible to estimate the newly infected retrospectively from the number of deaths) the basic reproduction number *R*_0_, which estimates the average number of infections generated by a person during the infectious period, is calculated using the average of the last 5 days with the estimated incidence, with the following formula:

$${{R_{0}=(E}_{t+1}-E}_{t}+\frac{E_{t}}{\lambda_{E}})(\lambda_{I}N/I_{t}S_{t})$$

Where *t* is the next to last day with information, *λ*_*E*_ the mean pre-infectious period, *λ*_*I*_ the mean duration of infection and *N* the total population.

The purpose of this methodology is to project the rate of exposed individuals and, consequently, the incidence, prevalence, and mortality of COVID-19 for a given period. The same procedure is used to project the transmission dynamics of the epidemic in the simulations that are presented by default in the interactive platform.

The source of the information on daily new infections and reported daily deaths is a dataset published daily by the European Center for Disease Prevention and Control [17].

In the case of the countries that are at the initial stages of the pandemic and with a low number of deaths, the number of infected subjects (I) was estimated from the reported cases, estimating a variable level of under-reporting according to the different testing-tracing strategies.

Table 1 describes the equations for the main model outputs in three different stages: stage A (from the initial day of the epidemic to the day of the beginning of the simulation minus 18 days), stage B (first 17 days of simulation), stage C (simulated period).

**Table 1:** Equations for the main model outputs in different stages.

Model outputs	Stage A	Stage B	Stage C
Daily new infected (*i*_*t*_)	$i_{t}=\frac{d_{t+17}}{f}$	$i_{t}=\frac{E_{t-1}}{\lambda_{E}}$	$i_{t}=\frac{E_{t-1}}{\lambda_{E}}$
Daily new deceases (*d*_*t*_)	*d*_*t*_ = *i*_*t*_*f*	$i_{t}=\frac{E_{t-1}}{\lambda_{E}}$	$i_{t}=\frac{E_{t-1}}{\lambda_{E}}$
Severe cases (*g*_*t*_)	*g*_*t*_ = *g*_*t*−1_+*i*_*t*−5_*s*−*i*_*t*−13_s where *s* = percentage of severe cases	*g*_*t*_ = *g*_*t*−1_+*i*_*t*−5_*s*−*i*_*t*−13_s where *s* = percentage of severe cases	*g*_*t*_ = *g*_*t*−1_+*i*_*t*−5_*s*−*i*_*t*−13_s where *s* = percentage of severe cases
Critical cases (*c*_*t*_)	*c*_*t*_ = *c*_*t*−1_+*i*_*t*−5_*u*−*i*_*t*−21_*u* where *u* = percentage of critical cases	*c*_*t*_ = *c*_*t*−1_+*i*_*t*−5_*u*−*i*_*t*−21_*u* where *u* = percentage of critical cases	*c*_*t*_ = *c*_*t*−1_+*i*_*t*−5_*u*−*i*_*t*−21_*u* where *u* = percentage of critical cases
Critical care beds used (*k*)	*k*_*t*_ = *c*_t_*d/*h* where *d* = days of hospitalization in the intensive care unit for critical cases and *h* = total days of hospitalization for critical cases	*k*_*t*_ = *c*_t_*d/*h* where *d* = days of hospitalization in the intensive care unit for critical cases and *h* = total days of hospitalization for critical cases	*k*_*t*_ = *c*_t_*d/*h* where *d* = days of hospitalization in the intensive care unit for critical cases and *h* = total days of hospitalization for critical cases
Ventilators used (*v*)	*v*_*t*_ = *k*_t_*j where *j* = number of estimated ventilators per critical care bed	*v*_*t*_ = *k*_t_*j where *j* = number of estimated ventilators per critical care bed	*v*_*t*_ = *k*_t_*j where *j* = number of estimated ventilators per critical care bed
Intensive care physicians (*m*)	*m*_*t*_ = *k*_t_*l where *l* = estimated number of physicians days per critical care bed	*m*_*t*_ = *k*_t_*l where *l* = estimated number of physicians days per critical care bed	*m*_*t*_ = *k*_t_*l where *l* = estimated number of physicians days per critical care bed
Nurses (*e*)	*e* = *k*_t_*o where *o* = estimated number of nurses day per critical care bed.	*e* = *k*_t_*o where *o* = estimated number of nurses day per critical care bed.	*e* = *k*_t_*o where *o* = estimated number of nurses day per critical care bed.

The epidemiological and clinical parameters used to simulate the initial scenario of each country came from rapid and "live" systematic bibliographic searches performed fortnightly, given the speed of appearance of new evidence in the initial stage of the pandemic.

A living targeted rapid systematic search was carried out through the main bibliographic databases as Medline, Embase, Scopus, Lilacs), Google Scholar, databases of preprints such as Medrxiv, Biorxiv, Arxiv, and ChinaXiv. This search was completed by including references used to populate main simulation models used globally, gray literature from generic search engines on the Internet, reference lists of relevant articles, specialized media, social networks and websites of various relevant scientific societies and organizations. The strategies used for the PubMed search (adapted for other databases) for each of the parameters are detailed in S1 Text. Specific criteria were defined for the selection of the relevant bibliography to select the key parameters to populate the model. These were the existence of a reproducible definition, a central estimate with its informed uncertainty (dispersion measures) from reliable sources (e.g., countries’ official records or recognized research bodies) and a high methodological quality, after applying an appropriate scoring tool for each type of study design. We prioritized data collected in primary research over simulations and reviews. Also, we considered their applicability to Latin America. Data was extracted in a template developed ad-hoc. The specific methodological aspects of the bibliographic review are described by Argento et al [18].

Information available on critical human and structural resources in each country (number of doctors and ICU doctors, ICU and hospital nurses, physiotherapists, ICU beds, and ventilators), was obtained through different sources of information. These sources include open government data, official COVID-19 monitoring boards, National Ministries of Health, Intensive Care Societies, Nursing Associations or Societies, Medical Associations, Societies and Colleges, Universities, among others. In addition, consultations with experts were performed to analyze the discrepancies, validate, and preselect the data. Thus, the final validation of the data was carried out through key informants selected by each country.

For the entry of the basic reproduction number by the user, which allows the simulation of scenarios of impact of different public policies, we used the model developed by the University of Oxford (http://epidemicforecasting.org/), to estimate the level of mitigation of R0 according to each policy applied and its level of compliance by the population.

The model runs on an interactive public access web platform using the statistical software R [19], through the Shiny framework [20], where the expected scenarios for 26 Latin American and The Caribbean countries can be accessed by any user. The default simulation scenario presented by the platform for each country is fed by the information available on incidence and mortality from COVID-19, the systematic review of clinical and epidemiological parameters, and the validated information on health resources for each country. Besides this default scenario, users can customize the model with personalized estimates that may better adjust to the epidemiological and health services context in each country at the time of interest.

The default simulation presented by the platform for each country is fed by the information available on incidence and mortality from COVID-19, the systematic review of clinical and epidemiological parameters, and the validated information on health resources for each country, previously mentioned.

Main model results can be visualized in the platform and include, among other secondary results: accumulated and daily numbers of new infected subjects and deaths, occupancy levels of ICU beds, ventilators, and number of critical human resources (ICU physicians and nurses).

The project also conveyed a scientific advisory group to provide inputs and feedback on the model results. The group members were selected for their expertise in health decision making, model building, infectious diseases epidemiology, intensive care, and hospital management. They provided advice on the potential impact of COVID‐19 epidemics and public health interventions on transmission dynamics and key health parameters. In addition, their advice allowed to validate the main model assumptions, the questions to be answered, and the general relevance. Furthermore, in the model design process, discussions were held with other modeling working groups such as "The COVID-19 Multi-model Comparison".

## Results

The model is publicly accessible through the following link: http://shinyapps.iecs.org.ar/modelo-covid19/. By default, the platform shows the results of an initial simulation of the selected country, based on the parameters collected through the systematic review of the literature and the SEIR model described above.

**Fig 1:**
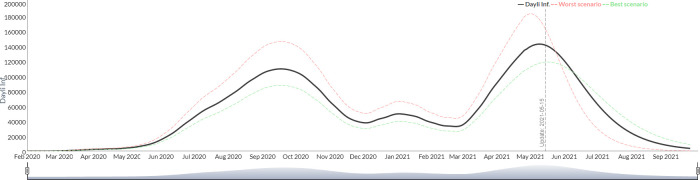
Simulation of the evolution of the new daily infected from July 2020 to September 2021 (Argentina).

To illustrate the results of the model, those from Argentina were selected from among the figures of the 26 countries included. The results are graphically presented on three scenarios: a base case scenario and an “optimistic" and "pessimistic" scenarios based on a percentage variation (customizable by the user) of a group of critical parameters. *The* infectious fatality rate (IFR) is the key parameter to derive the number of infected subjects and the percentage of patients requiring admission to a critical care unit as well as the length of stay in days in the critical care unit per patient. Fig 1 shows the simulation of the evolution of the new daily infected for Argentina from July 2020 to September 2020, using the described methodology.

The visualization of the trajectories in the same graph allows to evaluate two alternative dynamics of the epidemic, because the SEIR model culminates with 100% of the population infected (since it assumes that the entire population is susceptible). The configuration of three variable parameters (IFR, probability of severe case and percentage of critical care days) result in a scenario that reaches the peak in a shorter period accompanied with a steeper decline ("worse" scenario) and another with a slower distribution of infections over time determining a more flattened curve ("best” scenario).

**Fig 2:**
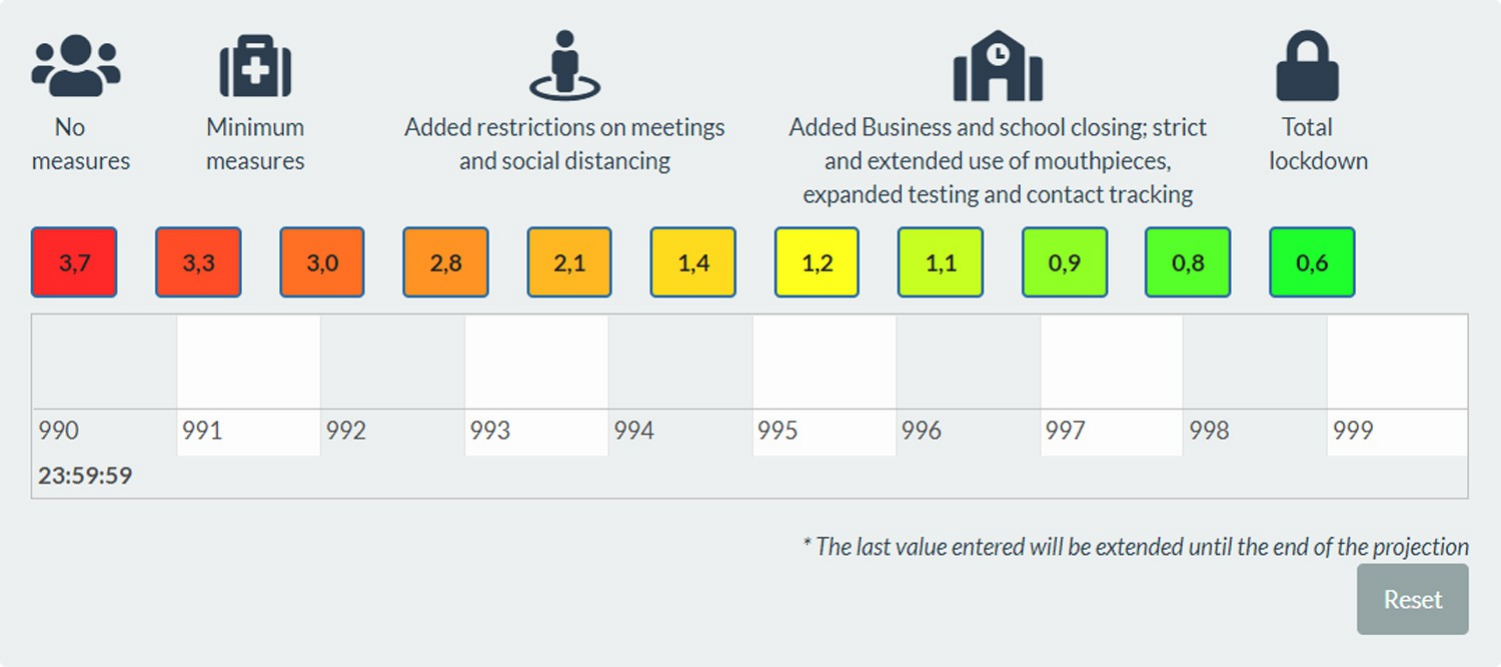
Traffic light of political interventions associated to R0 values.

Additionally, the user can visualize different scenarios that can be defined in three ways. 1) the first, called "Public Health Interventions" allows to establish the basic reproduction number (*R*_0_) on which the simulation will be based. This gradient of *R*_0_ are shown with a traffic light scale ranging from red (high *R*_0_) to green (low *R*_0_). One of the modalities contemplated to establish the *R*_0_ parameter based on the identification of intervention policies that are associated *a priori* to known *R*_0_ values in the bibliography, based on the *mitigation calculator for policymakers* from the University of Oxford [21]. The selected *R*_0_ may vary over the projected period, as shown in Fig 2.

**Fig 3:**
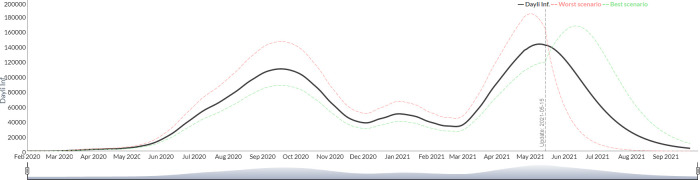
Simulation of the evolution of the new daily infected from July 2020 to September 2021 with constant R0 (Argentina).

**Fig 4:**
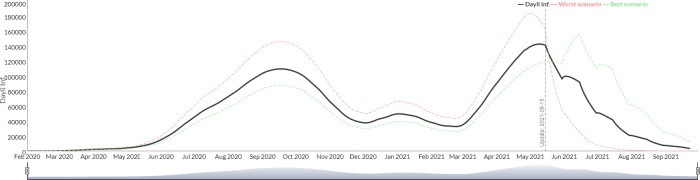
Simulation of the evolution of the new daily infected from July 2020 to September 2021 with oscilating R0 (Argentina).

**Fig 5:**
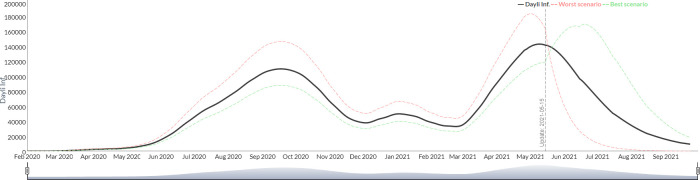
Simulation of the evolution of the new daily infected from July 2020 to September 2021 with reduction gradual intervention (Argentina).

**Fig 6:**
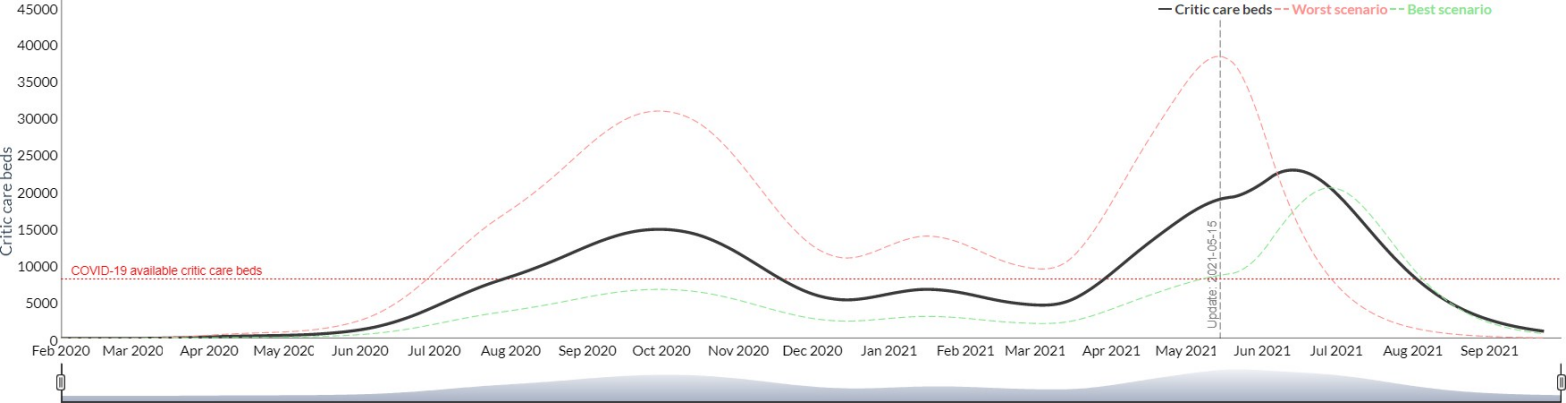
Simulation of the use of intensive care beds for Argentina based on a scenario of constant R_0 without intervention (Argentina).

**Fig 7:**
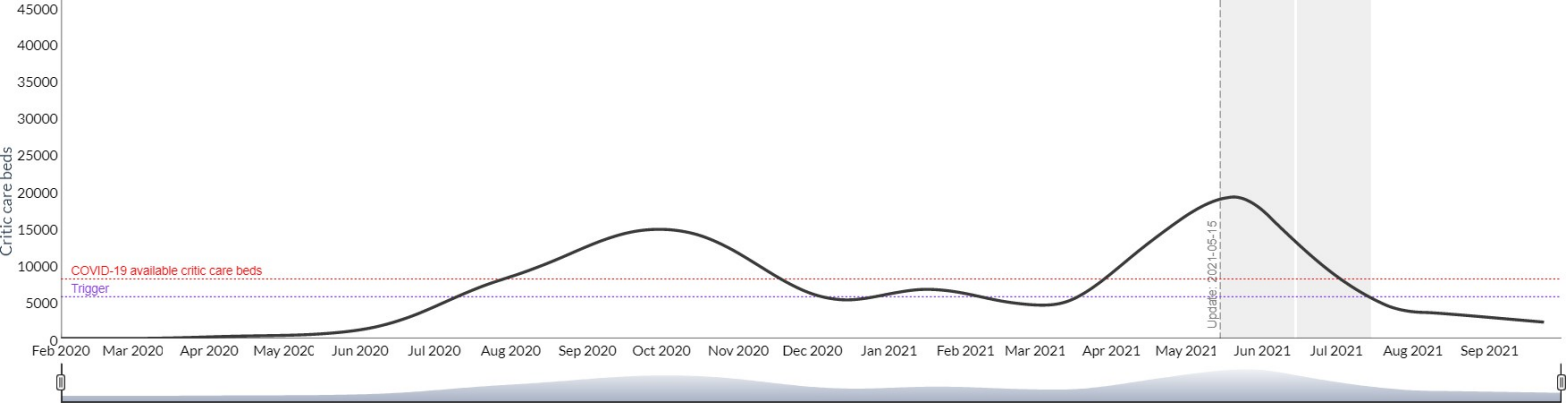
Simulation of the use of intensive care beds for Argentina based on a scenario of constant R_0 with a triggered intervention upon reaching 70% occupancy of intensive care beds (Argentina).

2) The second mode of setting scenarios in *"Public Health Interventions"* consists of the manual entry of *R*_0_ values for each period defined by the user. The configuration of the scenario can be simplified in this case by adopting predefined strategies: constant *R*_0_ with default policies unchanged throughout the entire projection), oscillating *R*_0_ (this resemble an intermittent valve where scenarios of hardness and flexibility of public health interventions, with their corresponding R0, are set between two values specified by the user, in configurable periods of time) and finally, gradual release of interventions (where the value of *R*_0_ is gradually increased from an initial value to a final value). Fig 3 shows the projected scenarios for Argentina under the simulation with a constant *R*_0_ of 2.3, Fig 4 shows intermittent valve oscillating between 1.9 and 2.5 every 15 days, and Fig 5 shows an scenario with reduction gradual intervention from an *R*_0_ of 2.3. In this example case it was observed that the first strategy predicted that 180 days after the forecast Argentina would accumulate 100530 deaths, while the second scenario 98957 and the last one 101189.

3) The R0 is established by defining a target intensive care bed occupancy rate that triggers more restrictive intervention policies. In this mode, the user must also enter the value of *R*_0_ expected from the intervention and for how long it would be maintained, and the output gives the user a projection of the dates and the number of restriction periods that would be required to maintain the ICU occupancy rate below the target. This option is of great interest for the projection of possible scenarios of health systems use and saturation. Figs 6 and 7 shows the simulation of the use of intensive care beds for Argentina based on a scenario of constant *R*_0_ of 2.8 without intervention and with a triggered intervention of *R*_0_ = 2 upon reaching 70% occupancy of intensive care beds. The same simulation can estimate the need of respirators, intensive care physicians and intensive care nurses.

The model platform provides the user with a summary of the main results to assess in each scenario set, since it estimates the dates when the highest number of new daily cases, deaths, maximum use of critical care beds and of ventilators will occur, with the corresponding values. It also estimates the number of days that have elapsed since -or will elapse- until those critical dates. The percentage of occupancy of critical beds and use of ventilators can also be observed.

In addition to the interactive visualization, the user can download a pdf file with the summary report of the parameters used, the results and the main graphics of the simulation.

## Discussion

In the present paper, we describe the process and main technical features of an open source, “user friendly” and publicly available model. This model was built through a collaborative project that involved an international team of public health researchers and decision makers, designed to be able to adequately depict the pandemic trajectory, and assess key policy questions in the 26 Latin America and The Caribbean countries. The model can help to respond to several key questions like a) how many total new infections and how many deaths are estimated per day?; b) when are the peaks of cases and deaths expected, based on the different scenarios?; c) what degree of occupancy rate will ICU services have along the epidemiological curve and the different scenarios?; d) to what extent will critical human resources, ICU beds or ventilators be used?; e) when is the optimal time to start a period of increased restrictions in order to prevent saturation of ICU beds?. The model not only projects the outcomes of the epidemic and health system capacity according to different scenarios; and mix and stringency of non-pharmacological interventions (NPIs) policies implemented, but also let local users “customize” its main parameters according to the specific context of the country, and fully interact with the model to improve local policy relevance and uptake. In addition, it also allows users to select a degree of intensity of local interventions and policies (traffic light with *R*_0_ depending on the stringency of the implementation for each public policy and each measure) which can be used at both national and sub-national level if there is a minimum set of epidemiological, policy and health resources data.

Several other models were developed to predict the trajectory of the pandemic in countries and sub-national areas. Multiple methodologies are commonly used for forecasting, such as probabilistic and stochastic models, machine learning, etc. The Institute for Health Metrics and Evaluation (IHME) projections are available online (https://covid19.healthdata.org/) and allow observing alternative scenarios in the evolution of the pandemic at a regional and country level. The platform includes projections of cases, deaths and the use of hospital and healthcare resources. For the projections of cases and deaths, the model allows multiple scenarios incorporating different levels of use of masks and variants in the vaccination campaigns. The Imperial College of the UK projects the total number of cases, the expected number of deaths following the infection, the number of people who will require mechanical ventilation and the impact of the public policies and interventions implemented by the countries. The model estimates scenarios at a national level adding age or income level segments or compartments (https://mrc-ide.github.io/). The OpenABM-Covid19 is a model developed by Oxford University to simulate the spread of the COVID-19 epidemic in cities, stratified by age and based on contacts between people. This model allows prediction of the impact contact tracing as a non-pharmaceutical intervention [22].

Additionally, we have undertaken a “living” rapid targeted systematic review to have the best set of parameters together with their uncertainty to better inform model parameters [23].

It should be noted that, although there are models that include predictions for Latin America and the Caribbean, they are not focused on the region and, do not include customized parameter estimates or scenarios to be used by analysts or decision makers.

Based on the possibility of modifying parameters by the user, the LA&C Health Systems Preparedness and Response Model for COVID-19 constitutes a valuable tool for simulating possible scenarios in the countries, considering expected dynamics in the maturation of the epidemic and potential government interventions. As an effect of the customization of key parameters, the model provides strategic results for decision-making by those who must manage public policies in this context: expected number of new infections and accumulated infections, expected number of deaths and accumulated deaths, use and occupancy rate of critical resources, both physical and human. On the other hand, the customization of the scenarios is carried out through a simple platform available online, oriented towards a user profile compatible with research analysts or decision makers.

Other models available online offer projections of critical variables for the evolution of the epidemic [24–26] such as the rate of reproduction, the speed of duplication of cases, incidence, mortality and variables of the demand for health systems. However, they do not offer customization that allow the projections to be adapted by defining the epidemiological parameters, the expected level of reproduction and the availability of resources. In this sense, the main difference between this model and those indicated above, lies in the fact that it constitutes a tool that is completely adaptable to a user who, based on the knowledge of the local context, can fine-tune it and adapt it to have better local estimates through a simple and intuitive user interface. It is important to highlight that the model is transparent for the user due to its accessible methodology and modifiable parameters.

A relevant element that arises from the review of available models is the treatment of uncertainty. Our model incorporates uncertainty in different ways. Besides the base-case scenario, we built two alternative simulations applying a variation of the parameters, resulting in a more favorable transmission dynamic and a less favorable one than the base case projection. Additionally, most of the parameters have a base-case value and a recommended uncertainty range (i.e 95% CI) and the user can assess deterministic scenarios with the best set of available local parameters. Other models [27,28] use probabilistic methodologies where multiple scenarios are simulated according to the estimated or reference distribution on relevant parameters, summarizing the trajectories in an expected range of values.

The main advantages of our model lie in its ability to predict configurable scenarios that are easy and quick to interpret for decision makers. It allows to establish the impact of the epidemic according to different scenarios and interventions implemented, and to anticipate when to intervene to avoid the collapse of critical services. On the other hand, it allows the parameterization of variables according to a specific context.

Additionally, being Open-Source, the source code is available in a public repository, the model can be customized for any population for which the necessary data is available, and can be executed on free on any computer. In this way, it is permanently available to technicians and decision-makers around the world, in a collaborative environment, where changes or improvements can be suggested and contributed.

The model also has some limitations. The real number of cases is estimated from the number of registered deaths and the *IFR* or a multiplier, which may represent errors and inaccuracies because the *IFR* values have considerable levels of uncertainty [29]. On the other hand, to derive total cases from deaths using IFR is more objective and transparent, not mentioning that it is better reported and less sensitive to local testing strategies. Another limitation is that interactions between people occur in the same way between all population groups and thus the overall fatality is the same in populations with different demographic structures but our model is currently incorporating age group compartments to the differential impact of vaccination strategies on the epidemic trajectory in countries in the region. In addition, the next versions of the model will also be subjected to calibration processes, which have been postponed in the first versions, more oriented to provide accessible tools for decision making.Another element to consider is that there may be different criteria in the countries regarding the determination of the cause of death in epidemiological surveillance systems.

It is important to emphasize that the aim of the model was not to accurately predict the course of the epidemic in a country, or the detailed impact of possible specific interventions (eg, to predict the local effect of lockdowns, opening or closing schools, restaurants, etc.), but to provide decision makers with different scenarios according to policies in different stages of the epidemic.

## Conclusion

In conclusion, our user friendly, transparent, and interactive model was developed to facilitate policy makers’ decisions, by allowing the user to modify its parameters according to the specific pandemic trajectory and policy context in each country, and therefore to ‘adjust’ the potential impact of public health interventions. Epidemiological models play a fundamental role in decision-making and public policies implementation, evaluation, and monitoring. The LA&C Health Systems Preparedness and Response Model to COVID-19 is a tool that frames scientific knowledge, empirical evidence and public policies in a fully transparent framework.

## Supporting information

S1 Text Bibliographic search strategies. (DOCX)
