# Supplementary material for: Development and application of a dynamic transmission model of health systems’ preparedness and response to COVID-19 in twenty-six Latin American and Caribbean countries
Source: PLOS Glob Public Health. 2022 Mar 8;2(3):e0000186. doi: 10.1371/journal.pgph.0000186 (PMC10021760; doi:10.1371/journal.pgph.0000186)
Supplement: S1 Text — (DOCX) [file pgph.0000186.s001.docx]

**S1. Bibliographic search strategies**

**Incubation period**

(Coronavirus[Mesh] OR Spike protein, SARS-CoV-2 [Supplementary Concept] OR Severe Acute Respiratory Syndrome Coronavirus 2[Supplementary Concept] OR COVID-19[Supplementary Concept] OR Corona Virus[tiab] OR COVID-19[tiab] OR COVID19[tiab] OR 2019-nCoV[tiab] OR SARS-CoV-2[tiab] OR SARS-CoV2[tiab] OR (Pneumonia[tiab] AND Wuhan[tiab] AND 2019[tiab]) OR (Coronavir*[tiab] AND 2019[tiab])) AND ((incubation [tiab] OR incubat*[tiab])AND period [tiab] AND (("2020/01/01"[PDAT] : "3000/12/31"[PDAT]) AND "humans"[MeSH Terms])

**Period of infection**

(Coronavirus[Mesh] OR Spike protein, SARS-CoV-2 [Supplementary Concept] OR Severe Acute Respiratory Syndrome Coronavirus 2[Supplementary Concept] OR COVID-19[Supplementary Concept] OR Corona Virus[tiab] OR COVID-19[tiab] OR COVID19[tiab] OR 2019-nCoV[tiab] OR SARS-CoV-2[tiab] OR SARS-CoV2[tiab] OR (Pneumonia[tiab] AND Wuhan[tiab] AND 2019[tiab]) OR (Coronavir*[tiab] AND 2019[tiab])) AND ((Transmission [tiab] OR contag*[tiab])AND period [tiab] AND (("2020/01/01"[PDAT] : "3000/12/31"[PDAT]) AND "humans"[MeSH Terms])

**Basic reproduction number (R0)**

Coronavirus[Mesh] OR Spike protein, SARS-CoV-2 [Supplementary Concept] OR Severe Acute Respiratory Syndrome Coronavirus 2[Supplementary Concept] OR COVID-19[Supplementary Concept] OR Corona Virus[tiab] OR COVID-19[tiab] OR COVID19[tiab] OR 2019-nCoV[tiab] OR SARS-CoV-2[tiab] OR SARS-CoV2[tiab] OR (Pneumonia[tiab] AND Wuhan[tiab] AND 2019[tiab]) OR (Coronavir*[tiab] AND 2019[tiab])) AND (Basic Reproduction Number[Mesh] OR Reproduction Rat*[tiab] OR Basic Reproduct*[tiab] OR R0[tiab] OR R [tiab] OR New Cases[tiab])

**Length of hospital stay**

(Length of Hospital Stay OR length of stay OR Hospitalization OR Hospitalization stay OR "Length of Stay"[Mesh]) AND (Coronavirus[Mesh] OR Spike protein, SARS-CoV-2 [Supplementary Concept] OR Severe Acute Respiratory Syndrome Coronavirus 2[Supplementary Concept] OR COVID-19[Supplementary Concept] OR Corona Virus[tiab] OR COVID-19[tiab] OR COVID19[tiab] OR 2019-nCoV[tiab] OR SARS-CoV-2[tiab] OR SARS-CoV2[tiab] OR (Pneumonia[tiab] AND Wuhan[tiab] AND 2019[tiab]) OR (Coronavir*[tiab] AND 2019[tiab]))

**Length of hospitalization in intensive care unit (ICU)**

(Length of intensive care unit stay OR length of ICU stay OR intensive care stay OR "Instensive care unit length of stay"[Mesh]) AND (Coronavirus[Mesh] OR Spike protein, SARS-CoV-2 [Supplementary Concept] OR Severe Acute Respiratory Syndrome Coronavirus 2[Supplementary Concept] OR COVID-19[Supplementary Concept] OR Corona Virus[tiab] OR COVID-19[tiab] OR COVID19[tiab] OR 2019-nCoV[tiab] OR SARS-CoV-2[tiab] OR SARS-CoV2[tiab] OR (Pneumonia[tiab] AND Wuhan[tiab] AND 2019[tiab]) OR (Coronavir*[tiab] AND 2019[tiab]))

**Percentage of inpatients requiring ICU**

Coronavirus[Mesh] OR Spike protein, SARS-CoV-2 [Supplementary Concept] OR Severe Acute Respiratory Syndrome Coronavirus 2[Supplementary Concept] OR COVID-19[Supplementary Concept] OR Corona Virus[tiab] OR COVID-19[tiab] OR COVID19[tiab] OR 2019-nCoV[tiab] OR SARS-CoV-2[tiab] OR SARS-CoV2[tiab] OR (Pneumonia[tiab] AND Wuhan[tiab] AND 2019[tiab]) OR (Coronavir*[tiab] AND 2019[tiab])) AND ("Intensive Care Units"[Mesh] OR ICU[tiab] OR Intensive Care Unit[tiab] OR "Critical Care"[Mesh] OR critical unit[tiab])

**Percentage of patients in ICU requiring mechanical ventilation**

Coronavirus[Mesh] OR Spike protein, SARS-CoV-2 [Supplementary Concept] OR Severe Acute Respiratory Syndrome Coronavirus 2[Supplementary Concept] OR COVID-19[Supplementary Concept] OR Corona Virus[tiab] OR COVID-19[tiab] OR COVID19[tiab] OR 2019-nCoV[tiab] OR SARS-CoV-2[tiab] OR SARS-CoV2[tiab] OR (Pneumonia[tiab]) AND ("Respiration, Artificial"[Mesh] OR Mechanical Ventilation[tiab] OR invasive ventilation[tiab])

**Fatality rate among infected**

Coronavirus[Mesh] OR Spike protein, SARS-CoV-2 [Supplementary Concept] OR Severe Acute Respiratory Syndrome Coronavirus 2[Supplementary Concept] OR COVID-19[Supplementary Concept] OR Corona Virus[tiab] OR COVID-19[tiab] OR COVID19[tiab] OR 2019-nCoV[tiab] OR SARS-CoV-2[tiab] OR SARS-CoV2[tiab] OR (Pneumonia[tiab] AND Wuhan[tiab] AND 2019[tiab]) OR (Coronavir*[tiab] AND 2019[tiab])) AND ((mortality [Mesh] OR mortality [tiab] OR death* [tiab]) AND ((infect* [tiab] AND fatality rate [tiab]) OR IFR OR “infection fatality rate”) AND (("2020/01/01"[PDAT] : "3000/12/31"[PDAT]) AND "humans"[MeSH Terms])
